# Tailorable Energy Absorption During Quasi-Static Crush via Additively Manufactured Honeycomb

**DOI:** 10.3390/polym17081050

**Published:** 2025-04-12

**Authors:** Colleen M. Murray, Grace N. Johnson, Min Mao, Norman M. Wereley

**Affiliations:** Composites Research Laboratory, Department of Aerospace Engineering, University of Maryland, College Park, MD 20742, USA; cmmurray@umd.edu (C.M.M.); graciej@umd.edu (G.N.J.); mmao@umd.edu (M.M.)

**Keywords:** honeycomb, energy absorption, buckling initiators

## Abstract

Honeycomb materials are being used for energy absorption applications in aerospace and automotive industries due to their high strength-to-weight ratio. In this work, additively manufactured honeycombs with different inscribed diameters were tested in quasi-static compression on a servo-hydraulic material test system to determine how the geometry affects the energy absorption properties. Samples were made from acrylonitrile butadiene styrene in order to study the performance of brittle honeycomb structures in energy absorption applications. Samples were manufactured with buckling initiators, or small triangle cutouts, located at varying distances from the bottom of the sample, while others had no modifications. These buckling initiators create preferential stress concentrations, thus encouraging a controlled folding of the structure. As this study shows, the crush efficiency and energy absorbed efficiency increase as the inscribed diameter decreases. When the inscribed diameter is 20 mm, the crush efficiency is 20.29%, while it is three times larger when the inscribed diameter decreases to 10 mm (62%). The energy absorbed efficiency is 45% for the 10 mm sample while it decreases to 16.70% when the diameter is 20 mm (a 36% decrease). Similarly, the presence of buckling initiators increases crush efficiency and energy absorbed efficiency when compared to samples of similar geometry but no buckling initiators, regardless of the size of the honeycomb.

## 1. Introduction

Additive manufacturing (AM) has been incorporated into industries such as automotive, construction, and aerospace in recent years [1]. The reduction in cost and time to manufacture complex geometries, along with minimal capital investment and versatile production, has greatly increased interest in AM [1,2]. The most common and affordable additive manufacturing technique is fused deposition modeling, or FDM [3,4]. Here, an object is manufactured by depositing the material layer by layer on a build plate [1]. The concern with this method is that the part will have high anistropy and weak interlayer bonding due to the material cooling in between layers [3,5].

The aerospace, automotive, and defense industries have become increasingly interested in the use of additively manufactured honeycomb (HC) for sandwich panels in energy absorption applications [6,7]. FDM can be used to print HC samples such that the HC cell longitudinal axis is perpendicular to the print plane, and when these samples are loaded in the out-of-plane direction, these samples exhibit much greater strength and energy absorption in the out-of-plane direction than in the in-plane direction. These HC core materials are of great interest due to their high strength-to-weight ratio, high stiffness-to-weight ratio, and high energy absorption capabilities [8,9,10]. As noted in [7], when designing an HC for energy absorption applications, the cell geometry must be designed in order to maintain transmitted force below the force threshold that may cause damage or injury.

The process for the manufacture of hexagonal honeycomb (HHC) structures is well known. For example, when manufacturing aluminum HHC, sheets of aluminum are stacked atop one another. Evenly spaced strips of adhesive are used to glue these sheets together, which alternate from layer to layer, so that when the stack of sheets is expanded (usually by inflation), the hexagonal cell shape is fabricated [11,12]. The spacing between these adhesive layers enables tailoring of the inscribed diameter of the cell. Each HHC cell varies in wall thickness around the perimeter because where adhesive is used to join the stacked sheets, bringing together two pieces of metal at the same location, a double wall thickness results. In contrast, manufacturing these honeycomb cores using FDM allows for greater control of HC cell geometry including inscribed diameter, a constant or varying wall thickness around the perimeter or along the longitudinal axis of the HHC cell, and shape (i.e., non-hexagonal HC cells such as circular, rectangular, or triangular HC cells). Thus, additive manufacturing enables HC cell geometry to be tailored to create HC structures that can be tailored to specific requirements [13].

Trigger mechanisms can be incorporated into energy absorbing structures to improve crashworthiness characteristics. These triggers decrease the peak stress and/or increase the mean crush stress so that these stresses are equilibrated [14]. Buckling initiators are the most common trigger mechanism, and these are also investigated here [15,16,17]. Buckling initiators are purposeful stress concentrators (such as a perforation in HHC wall) introduced into a structure to encourage failure [12,18,19]. Buckling initiators encourage failure at their given location, initiating folding in the structure walls [20,21,22]. These buckling initiators have been extensively studied in samples with circular and square cross-sections [16,17,23,24]; however, there are some examples of buckling initiators in honeycomb [25,26,27]. In our prior work [27], we conducted a similar study to this one in which visco-elastic HHCs were fabricated from a soft visco-elastic elastomer to to assess the efficacy of buckling initiators and showed that buckling initiators increased crush efficiency. However, the application of buckling initiators to increase efficiency in brittle plastic (such as ABS plastic) HHC has not been adequately investigated.

Thus, a second motivation arises for using additive manufacturing. In this study, we introduce buckling initiators (BIs) into the HHC structure to improve crashworthiness characteristics. In our case, we are considering diamond-shaped cutouts placed at the vertices of the HHC walls. Such a BI pattern can be readily fabricated using additive manufacturing. To introduce such BIs in a conventional metallic HHC would involve machining holes at the edges of the adhesive strips discussed above, and these holes would introduce residual stresses and create complications, with both adhesive possibly filling the BIs, and/or introducing complications to the HHC inflation process.

In this study, a series of HHC samples were tested under quasi-static compression with and without buckling initiators (BIs). Each of these honeycombs had a different inscribed diameter, increasing from 10 to 20 mm in increments of 5 mm. As the inscribed diameter increased, the crush efficiency was found to decrease; however, the samples with buckling initiators always outperformed their counterparts without buckling initiators. Manipulating these factors allows the ability to tailor the energy absorption properties for a given application.

This paper begins with a description of the process of manufacturing and testing samples in Section 2.1, followed by the metrics used for analysis in Section 2.2, and the methodology for computational analysis in Section 2.3. Section 3 details the results obtained by experimental testing (Section 3.1) prior to validating the experimental results in Section 3.2, and tailoring the results to fit certain safety criteria in Section 3.3. This paper ends with a discussion of the results and conclusions in Section 4 and Section 5, respectively.

## 2. Materials and Methods

### 2.1. Manufacturing and Testing

**Figure 1:**
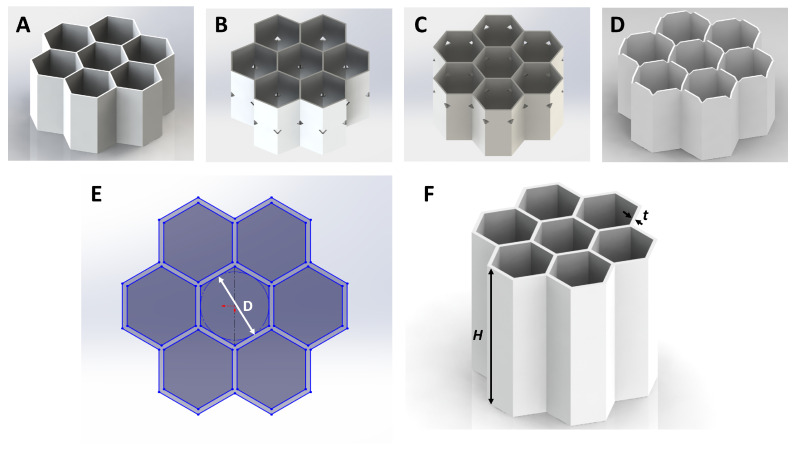
Computer -aided design rendering of samples. (**A**) 0 BI; (**B**) 0.50 BI; (**C**) 0.75 BI; (**D**) 1.00 BI; (**E**) inscribed diameter varies; (**F**) height and thickness of the sample are held constant.

For this study, a series of additively manufactured honeycombs of varying inscribed diameters were tested to identify the energy absorption properties. These samples were manufactured using acrylonitrile butadiene styrene (ABS-R), which has a tensile strength and modulus of 41 and 2400 MPa, respectively (Makerbot, New York, NY, USA, accessed 17 February 2025). Our samples were composed of six hexagons surrounding a central hexagon. For the study, three types of samples were manufactured: those with inscribed diameters of 10, 15, or 20 mm, respectively. The samples kept all other geometric values constant using a height of 30 mm and a wall thickness of 0.7 mm. Each type of sample was broken into four subgroups: no buckling initiators (0 BI), buckling initiators located halfway up the height (0.50 BI), buckling initiators located 3/4 up the height (0.75 BI), and buckling initiators located at the top of the sample (1.00 BI). The buckling initiators in this study were diamond cutouts located at the vertices of the samples to encourage a purposeful folding. These buckling initiators were 4 mm in length and 4 mm in height. This created a total of 12 different types of HHC samples (Figure 1).

The samples were designed using Solidworks and sliced using CloudPrint from Makerbot. A Method X FDM printer (Makerbot, New York, NY, USA) was used to print the samples. ABS-R was selected as the filament due to its material properties.

The samples were tested in uniaxial compression on a servo-hydraulic material test system (MTS, Eden Prairie, MN, USA). The samples were tested at a strain rate of $1\times 10^{-3}$ s^−1^ which corresponds to a cross-head velocity of 0.03 mm/s. The samples were imaged using a 35 mm digital camera every 10 s, so that failure mechanisms could be documented and reviewed after testing.

### 2.2. Metrics

Energy absorption metrics were identified from the literature to assist in the evaluation of these samples. The stress must first be determined.

(1) $$\sigma =\frac{2F}{21L^{2}\sqrt{3}}$$

Here, σ is the stress, *F* is the force, and *L* is the length of the side wall. Because the sample is composed of seven independent hexagons, the area was determined using the following equation:

(2) $$A=\frac{21\sqrt{3}}{2}L^{2}$$

From this, the energy absorbed can be determined.

(3) $$U\left(\epsilon \right)=\int_{0}^{0.85}\sigma \left(\epsilon \right)d\epsilon \;$$

where ϵ is the strain. Using the energy absorbed, the crush efficiency can be compared with the idealized energy absorption.

(4) $$\eta_{C}\left(\epsilon \right)=\frac{U(\epsilon )}{max[\sigma (0),\sigma (\epsilon )]\epsilon }=\frac{\sigma_{mc}}{max[\sigma (0),\sigma (\epsilon )]}$$

In this equation, $\sigma_{mc}$ is the mean crush stress and it can be found using the following equation:

(5) $$\sigma_{mc}=\frac{U(\epsilon )}{\epsilon }$$

The mean crush stress provides a representative value of the stress throughout the entire strain range of the sample. When the energy absorbed is compared to the idealized energy absorbed throughout the strain range, the energy absorbed efficiency can be determined. Mathematically, this would be the following:

(6) $$\eta_{EA}\left(\epsilon \right)=\frac{U(\epsilon )}{max[\sigma (0),\sigma (\epsilon )]\epsilon }=\frac{U(\epsilon )}{max[\sigma (0),\sigma (\epsilon )]}$$

### 2.3. Computational Analysis

In order to investigate the honeycomb behavior under uni-axial quasi-static compression, a finite element method (FEM) model was developed to simulate the compression scenario in ABAQUS 2023.

The dimensions, cell shape, and overall geometry of the honeycomb, including buckling initiators and wall thickness, were represented using the shell elements model. To explore the advantages of honeycomb structures with buckling initiators, the FEM model of the structure without buckling initiators was also simulated. The shell element thickness of both models was set to 0.7 mm. Two plates (modeled as rigid bodies) were added to the honeycomb to represent the MTS test fixture and facilitate the load being applied. In the uniaxial compression simulation, the bottom and top plates were modeled as a general surface-to-surface contact with the honeycomb having a friction coefficient of 0.6. The friction coefficient was selected so that the computed stress under compression, in particular the energy absorbed efficiency, nominally agreed with the experimental results. The bottom plate was fixed and a displacement of 24 mm was applied to the top plate to mimic the test. The explicit dynamic solver in ABAQUS was adopted with a simulation time period of 0.02 s, and the compression displacement increased from 0 to 24 mm in this period.

The material properties of the ABS-R used in the experimental testing are a tensile strength and modulus of 41 and 2400 MPa (Makerbot, New York, NY, USA, accessed 17 February 2025). To have the data match in energy absorption efficiency values, effective properties were used in FEM analysis. The elastic modulus used for the computational analysis was 1702 MPa, with a Poisson’s ratio of 0.193, and a yield stress of 71.79 MPa. For simplicity, a linear isotropic hardening plastic model after yielding was used in the simulation with a yield stress of 71.79 MPa at a plastic strain of 0 and 82 MPa at a plastic strain of 0.3. In addition, shear failure with an initiation value of 1.4 was added in the analysis.

## 3. Results

### 3.1. Experimental Tests

**Figure 2:**
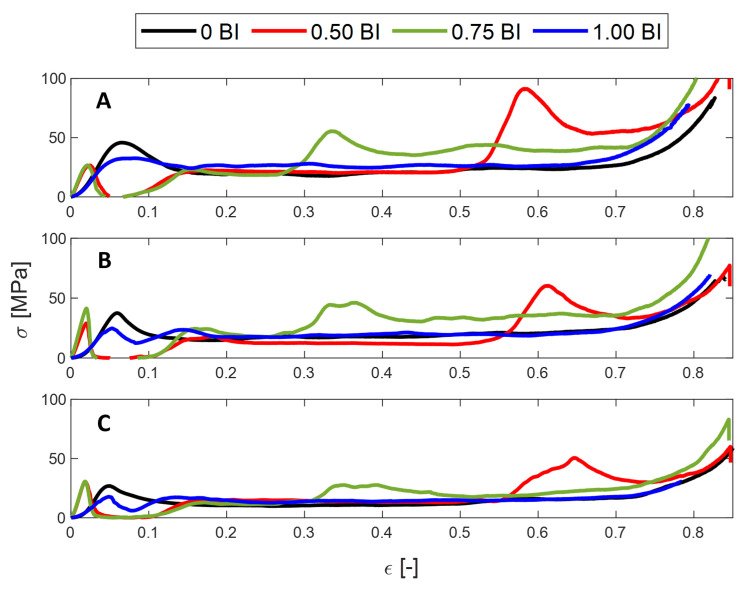
The stress vs. strain behavior of HHC samples under quasi-static conditions for different inscribed diameters: (**A**) 10 mm, (**B**) 15 mm, (**C**) 20 mm.

The peak stress is found to decrease as the inscribed diameter increases (Figure 2A–C). Because wall thickness is constant, the contact area over which the load is being applied increases with an increased inscribed diameter. For a constant load, stress will decrease as the cross-sectional area increases, as demonstrated in Figure 2A–C. In high-stress applications, such as impact events, a smaller inscribed diameter would be preferred because it can experience higher stress before deformation.

**Figure 3:**
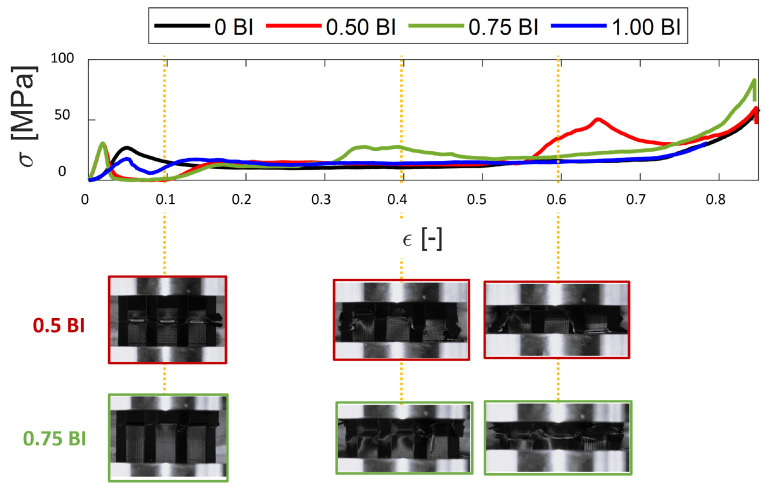
The deformation of the 20 mm samples with 0.50 BI and 0.75 BI.

As mentioned previously, the 0 BI samples do not have buckling initiators located at the bottom of the sample height, but, rather, do not have any buckling initiators at all. In contrast, the 1.00 BI samples have buckling initiators located at the top of the sample. Averaging across all inscribed diameters tested, the peak stress achieved by the 1.00 BI samples is about 10 MPa less than that achieved by the 0 BI samples (Figure 2A). As Figure 2 shows, the 0.50 BI and 0.75 BI samples exhibit a second peak in their respective stress–strain curves, therefore reducing their crush efficiencies when compared to both the 0 BI and 1.00 BI samples. This second peak is due to the location of the buckling initiators along the sample height, as shown in Figure 3. To assist in viewing this phenomenon, Figure 3 shows only the 0.50 BI and 0.75 BI samples with 20 mm inscribed diameters. Because 0 BI and 1.00 BI samples do not exhibit a second peak, photos of the deformation of these samples were not included in Figure 3. Once the buckling initiator region collapses, the stress will increase, generating a peak in the stress–strain curve. This pattern can be seen regardless of the inscribed diameter. As a result, having the buckling initiators at the top of the sample best equilibrates the peak stress with the mean crush stress, increasing crush efficiency.

**Figure 4:**
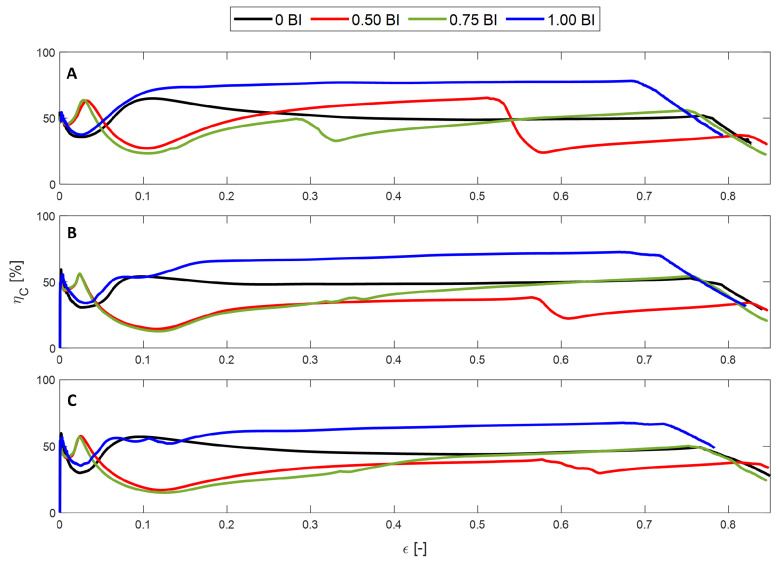
The strain dependent crush efficiency of HHC samples under quasi-static conditions for different inscribed diameters: (**A**) 10 mm, (**B**) 15 mm, (**C**) 20 mm.

The crush efficiency of these samples shows a consistent trend. The samples experience an initial increase in crush efficiency until they plateau before decreasing once densification begins, with the exception of the 0.50 BI sample. Figure 4A–C show that the maximum crush efficiency decreases as the inscribed diameter increases. Regardless of the inscribed diameter, the samples with the buckling initiators located at the top result in the highest crush efficiency across the usable strain range. This is followed by samples without buckling initiators for samples with inscribed diameters of 15 and 20 mm. When the inscribed diameter is smaller, having buckling initiators is found to increase the crush efficiency across the usable strain range.

Samples with 0.50 BI demonstrate a different crush efficiency behavior. The samples begin to increase in crush efficiency until 55–60% strain. At this point, there is a drastic knee, where the crush efficiency decreases by about 20%. This corresponds to the sudden increase in stress seen in Figure 3. Once 55% strain is reached, the calculation of the ideal energy absorbed now requires a stress value of 50 MPa as opposed to the previous 25 MPa, resulting in a drastic decrease in the crush efficiency value. It is important to note that there is a similar drop in the crush efficiency of the 0.75 BI sample in the 10 mm inscribed diameter sample. This corresponds, once again, to the sharp increase in the stress–strain curve. The other samples do not display this decline in crush efficiency because the secondary peak in stress does not surpass the initial peak stress value.

**Figure 5:**
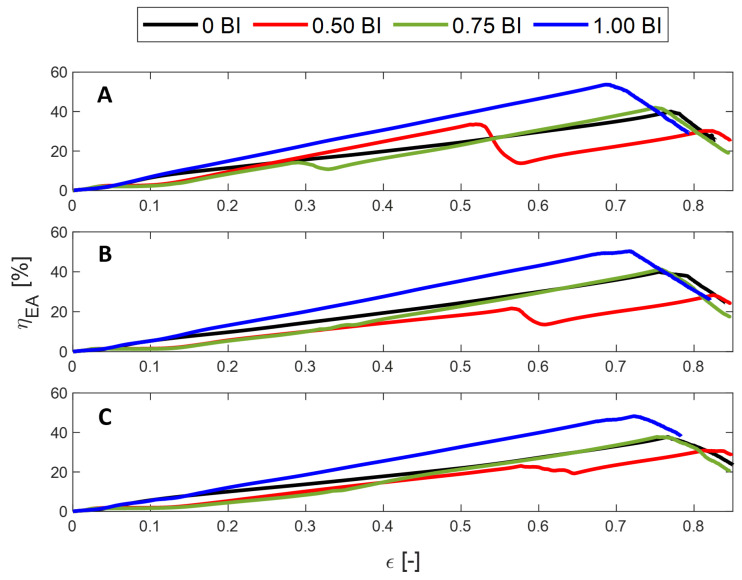
The energy absorbed efficiency of the samples under quasi-static conditions. (**A**) 10 mm, (**B**) 15 mm, and (**C**) 20 mm inscribed diameters.

The energy absorbed efficiency describes the ability of the sample to absorb energy, thus protecting the occupant. Regardless of the inscribed diameter, the maximum energy absorbed efficiency is achieved by the samples with buckling initiators located at the top, followed by 0 BI and 0.75 BI, depending on the cell size, and, finally, 0.50 BI (Figure 5). The samples also densify in the same order, so the 1.00 BI sample has the shortest usable strain range, up to about 70% strain compared to 0.50 BI, which is usable up to 85% strain.

Once again, there is a decline in the energy absorbed efficiency for the 0.50 BI samples and the 0.75 BI sample with a 10 mm inscribed diameter. This is a result of the increase in the maximum stress when computing the energy absorbed calculation. Because the contact area increases as the inscribed diameter increases, the energy absorbed efficiency is shown to decrease.

**Figure 6:**
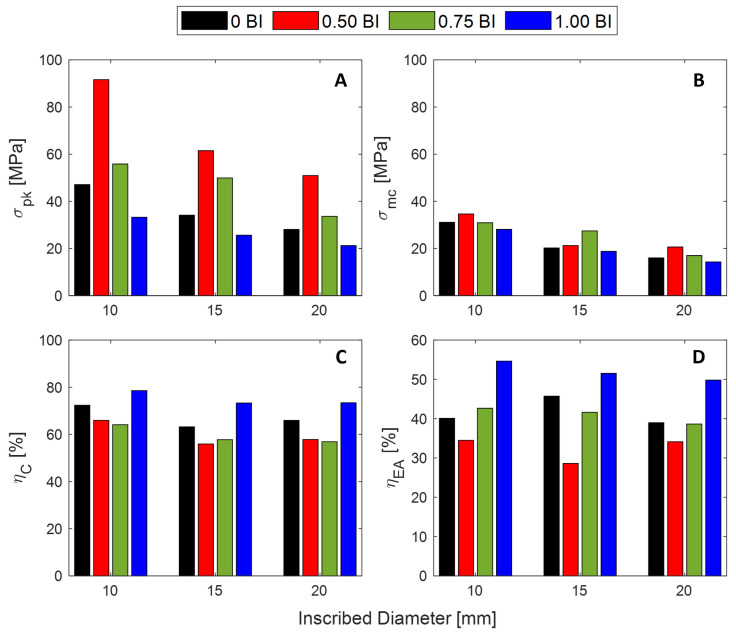
The metrics for the tested samples: (**A**) peak stress, (**B**) mean crush stress, (**C**) maximum strain dependent crush efficiency, and (**D**) maximum energy absorbed efficiency.

The metrics for these quasi-static tests are summarized in Figure 6. The peak stress (the maximum stress achieved prior to densification) is found to decrease as the height of the buckling initiator increases from the midpoint to the top of the sample, regardless of the inscribed diameter. The peak stress in the 10 mm inscribed diameter samples is shown to decrease from over 90 MPa to less than 40 MPa by moving the buckling initiator from the midpoint to the top of the sample (Figure 6A). In addition, increasing the inscribed diameter of the sample results in a decrease in the peak stress. For samples with the buckling initiator at the midpoint, the peak stress decreases from about 90 to 50 MPa by increasing the inscribed diameter from 10 mm to 20 mm.

The mean crush stress is a metric that reports the average stress value over the full strain range. According to this metric, the 0.50 BI sample has the highest average stress for the 10 and 20 mm inscribed diameter samples, closely followed by the 0.75 BI, which has the highest value for the 15 mm inscribed diameter samples (Figure 6B). The lowest reported mean crush stress for all samples is the 1.00 BI samples.

The maximum crush efficiency is highest for the 1.00 BI samples for all diameters tested by 5–10%. This is followed by samples without buckling initiators. Regardless of the situation, it will be more beneficial to use no buckling initiators before implementing buckling initiators 1/2 or 3/4 of the way up the sample.

The maximum energy absorbed efficiency is dominated by the 1.00 BI samples regardless of the sample diameter. These 1.00 BI samples are almost 10% more efficient than the next most efficient samples (Figure 6D). The quantitative values are provided in Table 1 below.

### 3.2. Validation of Results

#### 3.2.1. FEM Analysis

An FEM analysis was completed on the 0 BI and 1.00 BI samples to determine whether the experimental results could be predicted. These two classes of samples were selected because of their promising outcomes with regard to metrics.

**Figure 7:**
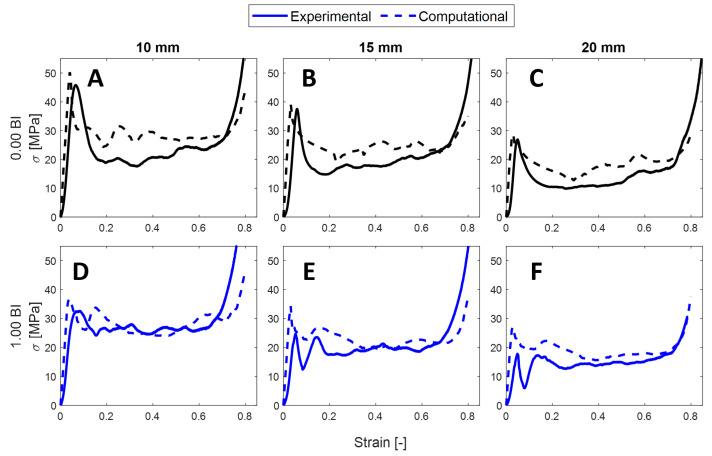
An FEM analysis of 0 BI samples (samples with no buckling initiators are plotted as black curves) and 1.00 BI samples (samples with buckling initiators at top are blue curves). (**A**) 10 mm with 0 BI; (**B**) 15 mm with 0 BI; (**C**) 20 mm with 0 BI; (**D**) 10 mm with 1.00 BI; (**E**) 15 mm with 1.00 BI; and (**F**) 20 mm with 1.00 BI.

The FEM analysis shows agreement with the experimental results for the 0 BI stiffness results. The initial increase in slope matches well, reaching a peak stress of similar values at the same strain (Figure 7A–C). The mean crush stress is overpredicted by the computational analysis by approximately 5 MPa in the three inscribed diameters. This will likely result in an overprediction of the crush efficiency.

**Figure 8:**
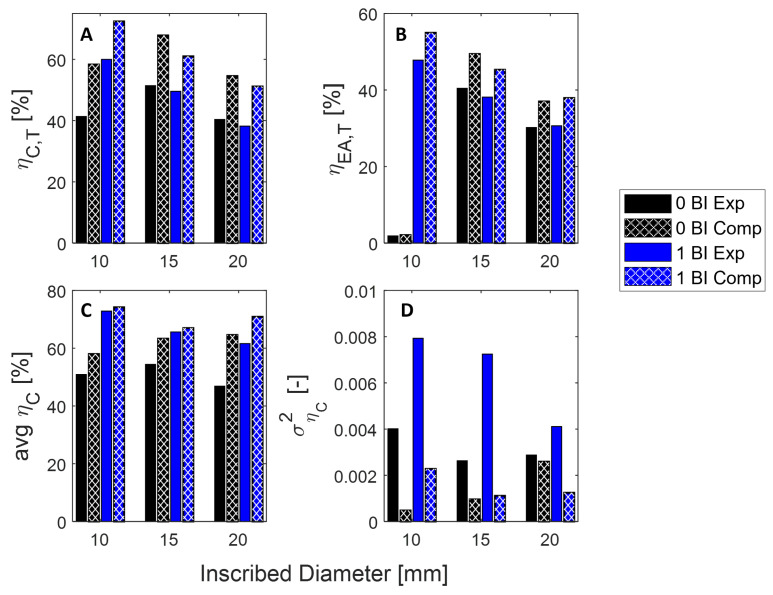
The FEM analysis metrics for the samples. (**A**) The threshold strain dependent crush efficiency (**B**) The threshold energy absorbed efficiency as calculated using a threshold of 40 MPa. (**C**) The average and (**D**) variance in crush efficiency from 2 to 75% strain. See Table A1 for numerical values.

Figure 8 shows the differences between metrics computed in our FEM model and those measured in experimental testing. Figure 8A–C show a general overprediction in maximum crush and energy absorbed efficiencies, and average crush efficiency, respectively. Despite this, the differences between these metrics when comparing the 0 BI case to the 1.00 BI case for a given inscribed diameter remain relatively consistent in both the experimental and FEM samples. For example, the average crush efficiency for 10 mm inscribed diameter samples increases by about 22% when going from the 0 BI case to the 1.00 BI case using our experimental data. Similarly, this metric increases by 16% in our FEM data.

The variance in this crush efficiency, shown in Figure 8D, is found to be significantly greater in the samples with buckling initiators when compared to samples without according to our experimental analysis. Conversely, for the 10 mm and 15 mm inscribed diameter samples, the FEM analysis suggests significantly less difference in the variance in crush efficiency between the 0 BI and 1.00 BI samples. Additionally, the FEM analysis suggests that 20 mm inscribed diameter samples with buckling initiators at the top will have less variance in crush efficiency than their 0 BI counterparts. With this in mind, this FEM analysis will still allow the ability to design future samples without extensive testing.

#### 3.2.2. Analytical Results

The analytical results for honeycomb materials have existed for well over 50 years. The original buckling load within a single panel of a honeycomb was derived by Timoshenko and Gere in 1961 [28]. In their work, Timoshenko et al. determined that the buckling load is

(7) $$P_{crit}=\frac{KE_{s}}{\left(1-v_{s}^{2}\right)}\frac{t^{3}}{l}$$

where *K* is a constant for the end constraint factor, $E_{s}$ is the modulus of the material, $v_{s}$ is the Poisson’s ratio, and the remaining variables correspond to geometric dimensions of the honeycomb. The honeycombs in this study have a singular wall thickness, *t*, so the elastic buckling stress must be slightly rederived, since Roark’s equation (originally derived from Timoshenko et al.) assumes walls of varying thickness [29].

For this study, the elastic buckling stress was derived knowing that all six walls have a singular thickness, *t*, and, thus, will maintain the same load after they reach a collapse load of $P_{crit}$ [29]. The elastic buckling load is the sum of the individual loads that each walls carries, so the elastic collapse stress or peak stress is

(8) $$\sigma_{3}=\frac{6P_{crit}}{2cos\alpha \left(1+sin\alpha \right)l^{2}}=\frac{3KE_{s}}{\left(1-v_{s}^{2}\right)cos\alpha \left(1+sin\alpha \right)}\frac{t^{3}}{l^{3}}$$

where α is the internal angle of the hexagon.

This equation requires knowing the material properties of the honeycomb. From the data sheets, the modulus was determined to be 2400 MPa with a yield stress of 41 MPa and a Poisson’s ratio of 0.25 (Ultimaker, New York, NY, USA). To determine the predicted value, it is important to identify the end constraint factor. When the ends are rigidly clamped, the literature has shown that K=6.2; however, this is not always the case [28,29]. A conservative estimate is K=3.29 because each wall is simply supported along the edges. These two values will provide an upper and lower bound, respectively, for the elastic collapse stress of the tested honeycomb [29]. To analyze the behavior of these honeycombs, the end constraint factor chosen for this analysis was K=5.73 because it is preferential to a clamped setup, but it is not rigidly clamped [28].

**Figure 9:**
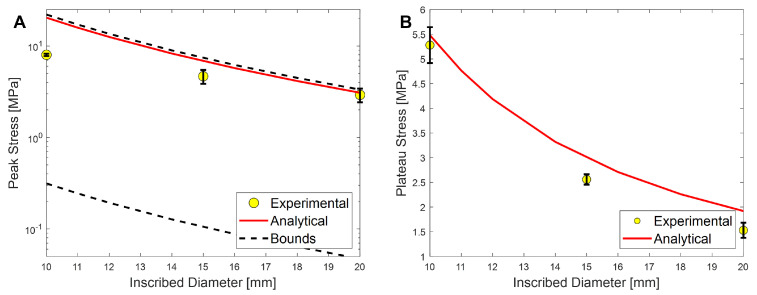
Analytical analysis of the experimental results for (**A**) peak stress and (**B**) plateau stress.

As Figure 9A shows, the experimental peak stresses fall within the upper and lower bounds that were derived using the Roark analysis. Each of these points is an average peak stress for the given inscribed diameter, with the error bar denoting the standard deviation of the measurements. The numerical values are presented in Table 2. The analytical analysis is an appropriate fit for this data, confirming the experimental tests.

Wierzbicki derived another relationship to describe the behavior of honeycomb. This determines the plastic buckling stress of honeycomb using the geometric and material properties [30]. Ashby and Gibson provide an overview of the derivation for regular hexagons with uniform wall thickness [31]. This plastic buckling stress or mean crush stress is defined as

(9) $$\sigma_{3}=5.6\sigma_{ys}{\left(\frac{t}{l}\right)}^{5/3}$$

This is the region within which the buckling occurs.

Figure 9B shows that the analytical equation is a good predictor of the mean crush stress of the experimental samples for the ABS-R honeycomb, again confirming the experimental results.

### 3.3. Tailoring to Safety Criteria

**Figure 10:**
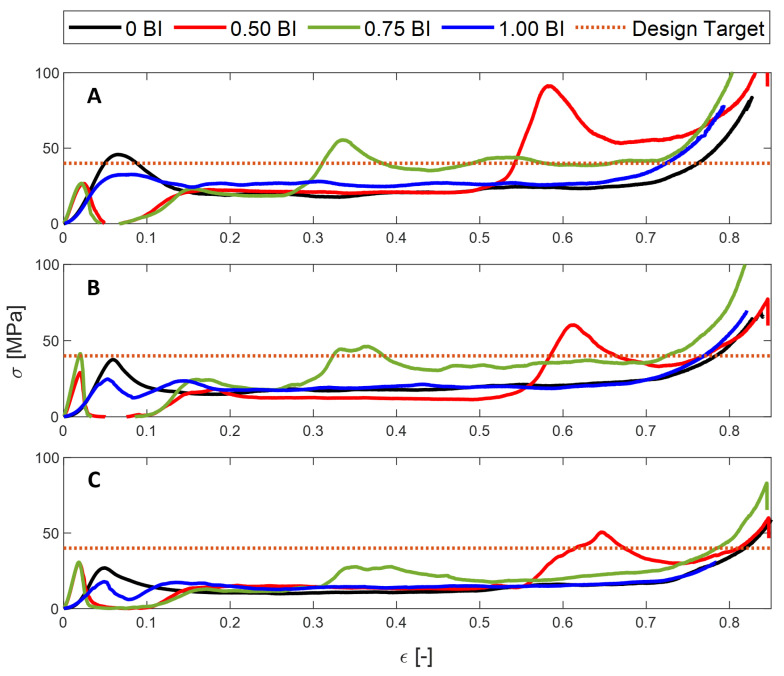
Analyzing the samples for a given design application using a representative threshold stress value of 40 MPa where (**A**) is 10 mm, (**B**) is 15 mm, and (**C**) is 20 mm inscribed diameter.

When designing a honeycomb structure for an end-use case, the stress the samples experience must not exceed a threshold stress value. At this point, the stress would cause damage to the occupant. For this study, the representative threshold stress selected was 40 MPa. Figure 10 shows the threshold stress marked on the stress–strain curves. Any of the samples that exceed this threshold stress are considered ineffective for the given application. Reviewing Figure 10A, 0 BI, 0.50 BI, and 0.75 BI cannot meet the threshold requirement. Similarly, 0.75 BI and 0.50 BI are unable to meet the threshold requirement for the 15 mm diameter samples, and the 0.50 BI sample is unable to meet the requirement for the 20 mm diameter samples.

The metrics must be calculated using a modified version of the crush and energy absorbed efficiency equations when being implemented in a design application. The crush efficiency equation is modified to always use a maximum stress value of the threshold stress:

(10) $$\eta_{C,T}\left(\epsilon \right)=\frac{U(\epsilon )}{\sigma_{T}\epsilon }=\frac{\sigma_{mc}}{\sigma_{T}}$$

where $\sigma_{T}$ is the threshold stress, and the mean crush stress ($\sigma_{mc}$) is the ratio of the energy absorbed over the strain. A similar modification is implemented in the energy absorbed efficiency equation:

(11) $$\eta_{EA,T}\left(\epsilon \right)=\frac{U(\epsilon )}{\sigma_{T}\epsilon }=\frac{U(\epsilon )}{\sigma_{T}}$$

It is important to remember that the strain is set equal to 1 when analyzing the energy absorbed efficiency because it is evaluating the efficiency over the entire strain range.

**Figure 11:**
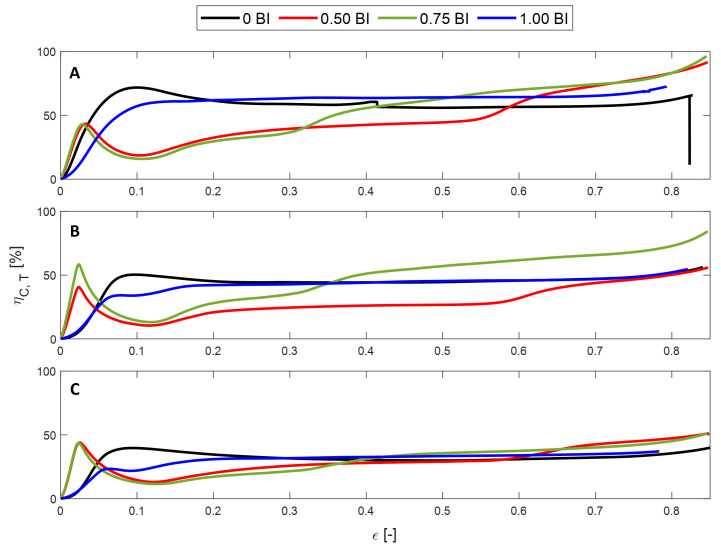
The crush efficiency of the quasi-static samples after the threshold has been applied for an inscribed diameter of (**A**) 10 mm, (**B**) 15 mm, and (**C**) 20 mm.

The crush efficiency shows the performance of these samples when taking into account the threshold stress of 40 MPa. In Figure 11, the 1.00 BI sample reaches a crush efficiency of 65% by 10% strain and maintains it over the full strain range. Similarly, at 15 mm, the 0 BI and 1.00 BI samples reach a consistent crush efficiency of 45% by 25% strain and hold that value until densification is achieved.

**Figure 12:**
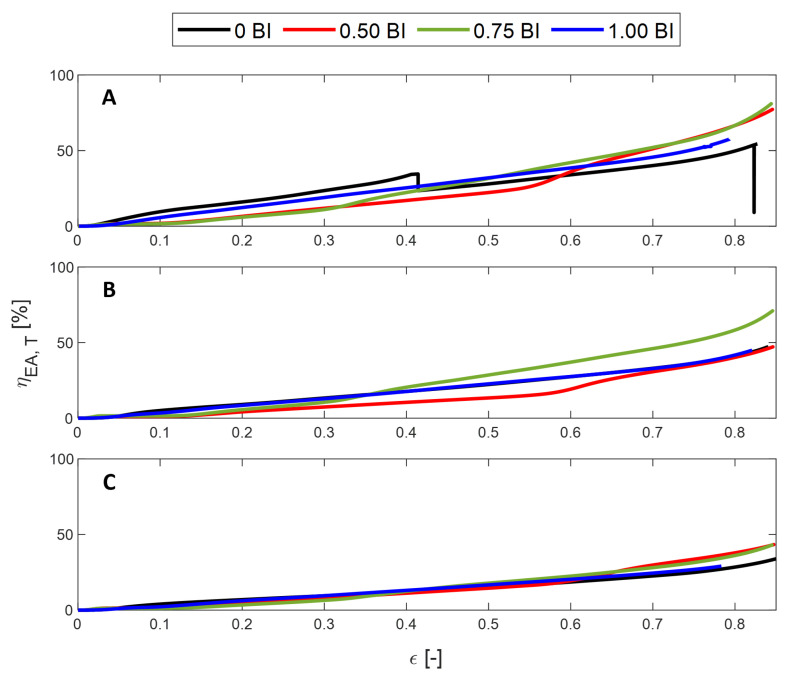
The energy absorbed efficiency of the samples tested in quasi-static conditions after the threshold has been applied. (**A**) 10 mm, (**B**) 15 mm, and (**C**) 20 mm inscribed diameters.

The energy absorbed efficiency is also plotted in Figure 12. For all samples, it is shown to be constantly increasing. As the inscribed diameter increases, the maximum energy absorbed efficiency is found to decrease. None of the samples are found to densify in the given strain range, either.

**Figure 13:**
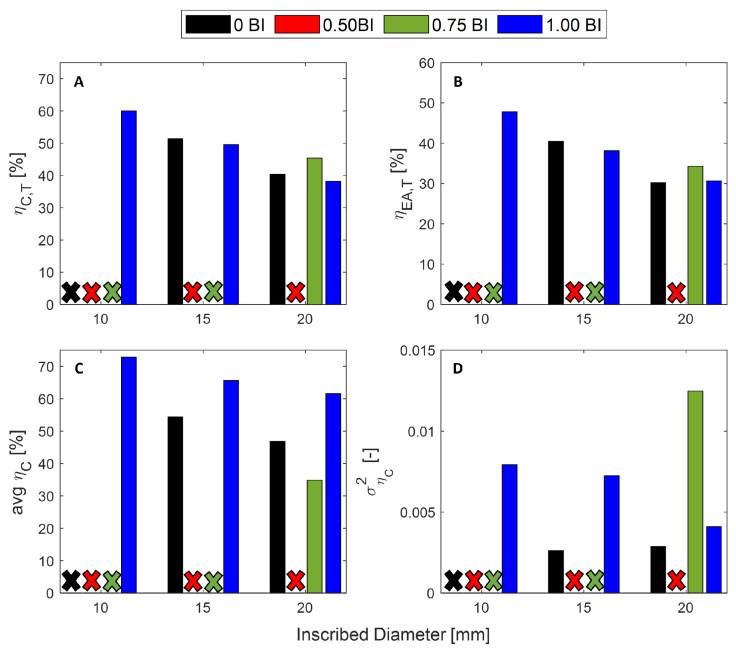
The design metrics as calculated using the threshold stress for (**A**) the threshold crush efficiency, (**B**) the threshold energy absorbed efficiency, (**C**) the average crush efficiency, and (**D**) the variance in crush efficiency from 2 to 75% strain. Any samples that had not met the threshold requirement are denoted with an ”×”.

Performance metrics are reported in Figure 13. Any sample that did not meet the threshold requirement is denoted by an “×” of the respective color. Although the maximum crush efficiency was the lowest for the 1.00 BI samples at all diameters, the average crush efficiency was the highest for these samples.

The energy absorbed efficiency and the variance of the crush efficiency further confirm why these 1.00 BI samples are a good candidate for this application.

## 4. Discussion

**Figure 14:**
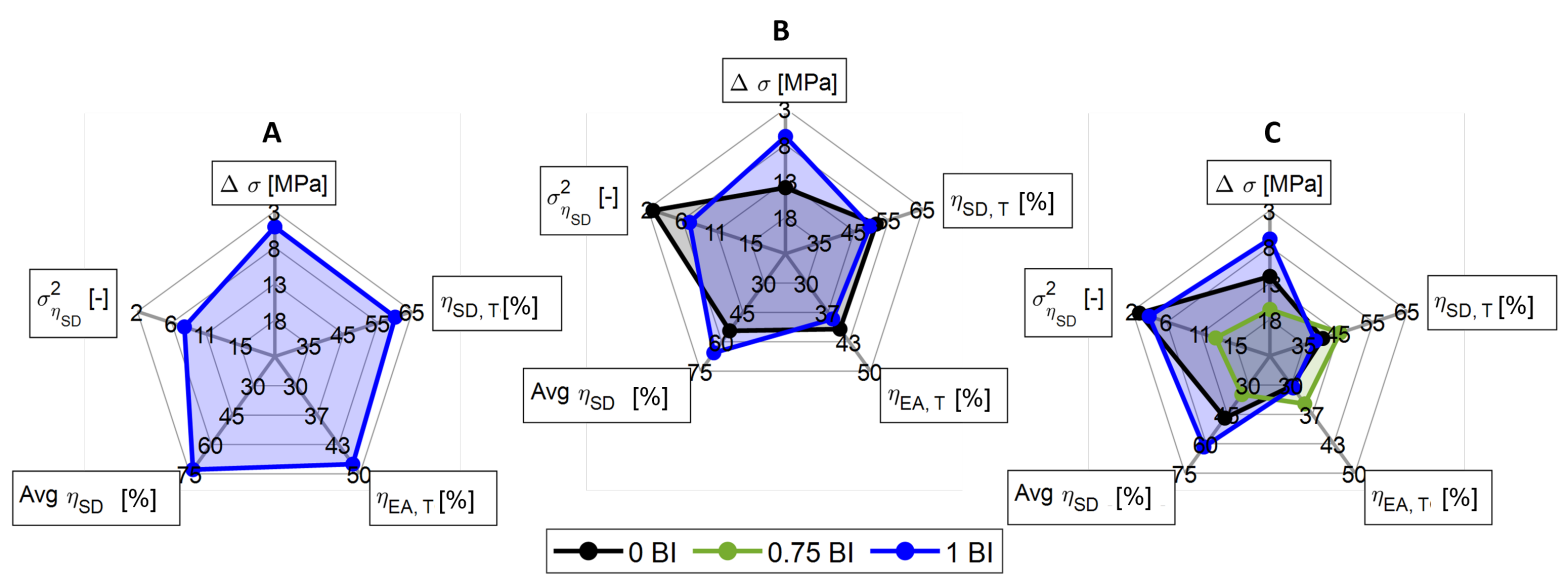
A radar plot of the samples that meet the threshold requirement for the design application for the inscribed diameters as follows: (**A**) 10 mm, (**B**) 15 mm, (**C**) 20 mm.

As discussed throughout this paper, the geometry of the honeycomb cell will affect the energy absorption properties. To demonstrate the effects of the selected metrics, a series of radar plots is presented in Figure 14 to aid in the decision-making process. Each spoke represents a different crashworthiness metric of interest. Beginning with the topmost spoke, the change in stress is the difference between the peak stress and the plateau stress, as shown in Table 3. This spoke has been inverted so that the smallest value (3 MPa) is on the exterior of the curve. The lower the deviation between the peak and plateau stress, the more area under the curve and, thus, an increase in crashworthiness. The next spoke represents the maximum crush efficiency recorded for the thresholded data. The spoke is designed to have the maximum crush efficiency on the exterior and the minimum crush efficiency shown on the interior. The third spoke analyzes a similar characteristic called threshold energy absorbed efficiency. Once again, it shows the maximum efficiency on the exterior of the radar and the minimum efficiency on the interior of the radar. The final two spokes return to analyzing the initial data without thresholding it. The first shows the average crush efficiency recorded between 2 and 75% strain. This is representative of the efficiency that the sample would see over a full impact event. Similarly, on the final spoke, the variance of the crush efficiency is provided. This is intended to provide additional information on the relative error seen with this sample.

Figure 14 shows all three geometries as radar plots. Each plot consists of the five metrics that have been studied throughout this work. The values most suited for energy absorption applications are listed furthest from the center of the radar plot. Each of these three plots shows that the 1.00 BI sample encompasses the entire 0 BI sample. This is indicative of the 1.00 BI sample outperforming the 0 BI in all metrics, regardless of the geometry of the cell. When looking at the individual radar plots, it is evident that the 1.00 BI sample with the smallest inscribed diameter (Figure 14A) is the best or second best choice in all metrics except the peak crush stress. For this reason, the 10 mm inscribed diameter honeycomb with buckling initiators will perform the best in crashworthiness applications.

## 5. Conclusions

During the course of this study, honeycombs with three different inscribed diameters were manufactured using ABS-R to study the influence of buckling initiators on changing geometries. The following conclusions can be drawn from this work.

1. The peak stress and the mean crush stress will decrease with increasing inscribed diameter, regardless of the presence of buckling initiators. Similarly, the crush and energy absorbed efficiency will decrease as the inscribed diameter increases. For all metrics, except for the peak stress, the presence of buckling initiators will increase the performance compared to their counterparts. As mentioned above, the peak stress will decrease with the presence of buckling initiators.
2. The performance of these samples was validated through the use of a finite element model in ABAQUS. The same trends are apparent: as the inscribed diameter increases, the peak and mean crush stress and energy absorbed and crush efficiencies will decrease regardless of the presence of buckling initiators.
3. The analytical analysis validated the performance of the samples as well. The re-derived version of Roark’s equation allows the peak stress to be accurately predicted. The peak and mean crush stress decrease with increasing inscribed diameters.

## Figures and Tables

**Table 1:** The average properties for the samples with varying BI location and inscribed diameters.

D	BI	$\sigma_{\mathrm{pk}}$	$\sigma_{\mathrm{mc}}$	Δσ	$\eta_{C}$	$\eta_{\mathrm{EA}}$
[mm]	[-]	[MPa]	[MPa]	[MPa]	[%]	[%]
10	0.00	47.09	31.09	16.00	72.37	40.10
	0.50	91.62	34.66	56.96	65.96	34.49
	0.75	55.83	30.92	24.92	64.11	42.65
	1.00	33.26	28.11	5.15	78.57	54.64
15	0.00	34.11	20.21	13.90	63.20	45.72
	0.50	61.48	21.25	40.24	55.92	28.60
	0.75	49.92	27.47	22.45	57.77	41.62
	1.00	25.63	18.77	6.86	73.31	51.53
20	0.00	28.10	16.02	12.08	65.92	38.97
	0.50	50.94	20.63	29.32	57.83	34.31
	0.75	33.64	17.02	16.62	56.90	38.64
	1.00	21.24	14.31	6.93	73.40	49.82

**Table 2:** The results of the various analytical models in comparison with the experimental results.

	Diamater	Upper Bound	Analytical	Experimental	Lower Bound
	[mm]	[MPa]	[MPa]	[MPa]	[MPa]
$\sigma_{pk}$	10	22.05	20.38	8.00	0.31
	15	6.84	7.41	4.66	0.13
	20	3.33	1.92	2.92	0.05
$\sigma_{pl}$	10	-	5.48	5.28	-
	15	-	2.99	2.56	-
	20	-	1.92	1.53	-

**Table 3:** The minimum and maximum range over which each individual spoke is plotted.

	Δσ	$\eta_{\mathrm{SD},T}$	$\eta_{\mathrm{EA},T}$	Avg $\eta_{\mathrm{SD}}$	$\sigma_{\eta_{\mathrm{SD}}}^{2}$
	[MPa]	[%]	[%]	[%]	[-]
**Minimum**	3	35	30	30	2
**Maximum**	18	65	50	75	15

## Data Availability

The original contributions presented in this study are included in the article. Further inquiries can be directed to the corresponding author.

## Abbreviations

AM	Additive Manufacturing
BI	Buckling Initiators
CE	Crush Efficiency
EA	Energy Absorption
FDM	Fused Deposition Modeling
HC	HoneyComb
HHC	Hexagonal HoneyComb
0 BI	HHC sample with no buckling initiators
0.5 BI	HHC sample with BI halfway up the sample
0.75 BI	HHC sample with BI three quarters of the way up the sample
1.0 BI	HHC sample with BI at the top of the sample

## Appendix A

The metrics were determined for both the experimental and computational results. The differences between these values are reported in the following table to provide additional insight into the precision of the model.

**Table A1:** The computational and experimental metrics for all three inscribed diameters.

Diameter			$\sigma_{\mathrm{pk}}$	$\sigma_{\mathrm{mc}}$	$\eta_{\mathrm{SD},T}$	$\eta_{\mathrm{EA},T}$	Avg $\eta_{\mathrm{SD}}$	$\sigma_{\eta_{\mathrm{SD}}}^{2}$
(mm)			(MPa)	(MPa)	(%)	(%)	(%)	(-)
10	0 BI	Measured	8.00	5.28	41.35	1.86	50.90	0.0040
		Computed	50.30	30.99	58.34	2.10	57.99	0.0005
		% Change	528.8	998.3	41.08	12.90	13.93	87.5
	1 BI	Measured	5.93	5.01	60.01	47.79	72.84	0.0079
		Computed	36.73	28.96	72.39	54.96	74.17	0.0022
		% Change	519.4	478.0	20.63	15.00	1.83	72.15
15	0 BI	Measured	4.66	2.84	51.40	40.44	54.40	0.0026
		Computed	39.13	27.13	67.82	49.41	63.32	0.0010
		% Change	739.7	855.3	31.95	22.18	16.40	61.59
	1 BI	Measured	3.38	3.10	49.57	38.15	65.62	0.0072
		Computed	34.32	24.40	60.99	45.30	66.98	0.0011
		% Change	915.4	687.1	23.04	18.43	2.07	84.72
20	0 BI	Measured	2.92	1.77	40.37	30.20	46.87	0.0029
		Computed	28.74	21.82	54.55	37.00	64.58	0.0026
		% Change	884.2	1132	35.13	22.52	37.78	10.34
	1 BI	Measured	2.27	1.72	38.21	30.64	61.61	0.0041
		Computed	27.08	20.46	51.16	37.92	70.88	0.0012
		% Change	1092	1089	33.89	23.76	15.05	70.73

## References

[1] Mansour M.T., Tsongas K., Tzetzis D. (2021). 3D Printed Hierarchical Honeycombs with Carbon Fiber and Carbon Nanotube Reinforced Acrylonitrile Butadiene Styrene. J. Compos. Sci..

[2] Joseph A., Mahesh V., Mahesh V. (2023). Effect of Loading Rates on the In-Plane Compressive Properties of Additively Manufactured ABS and PLA- Based Hexagonal Honeycomb Structures. J. Thermoplast. Compos. Mater..

[3] Domingues-Rodriguez G., Ku-Herrera J., Hernandez-Perez A. (2018). An Assesment of the Effect of Printing Orientation, Density, and Filler Pattern on the Compressive Performance of 3D Printed ABS Structures by Fuse Deposition. Int. J. Adv. Manuf. Technol..

[4] Yavuz G., Kiral B., Hizarci B., Kiral Z. (2022). Low- Velocity Single and Repeated Impact Behavior of 3D Printed Honeycomb Cellular Panels. Mater. Test..

[5] Es-Said O., Foyos J., Noorani R., Mendelson M., Marloth R., Pregger B. (2000). Effect of Layer Orientation on Mechanical Properties of Rapid Prototyped Samples. Mater. Manuf. Process..

[6] Ozen I., Cava K., Gedikli H., Alver U., Aslan M. (2020). Low-Energy Impact Response of Composite Sandwich Panels with Thermoplastic Honeycomb and Reentrant Cores. Thin-Walled Struct..

[7] Habib F., Iovenitti P., Masood S., Nikzad M. (2018). Cell Geometry Effect on In-Plane Energy Absorption of Periodic Honeycomb Structures. Int. J. Adv. Manuf. Technol..

[8] Menegozzo M., Cecchini A., Just-Agosot F., Acevedo D., Velez O., Acevedo-Figueroa I., Ruiz J. (2022). A 3D Printed Honeycomb Cell Geometry Design with Enhaced Energy Absorption under Axial and Lateral Quasi-Static Compression Loads. Appl. Mech..

[9] Gordelier T., Thies P., Turner L., Johanning L. (2019). Optimising the FDM Additive Manufacturing Process to Achieve Maximum Tensile Strength: A State-of-the-Art Review. Rapid Prototyp. J..

[10] Raut S., Jattie V., Khedkar N., Singh T. (2014). Investigation of the Effect of Built Orientation on Mechanical Properties and Total Cost of FDM Parts. Procedia Mater. Sci..

[11] Ivanez I., Fernandez-Canadas L., Sanchez-Saez S. (2017). Compressive Deformation and Energy Absorption Capability of Ability of Aluminum Honeycomb Core. Compos. Struct..

[12] Zhang X., Su H., Yu T. (2009). Energy Absorption of an Axially Crushed Square Tube with a Buckling Initiator. Int. J. Impact Eng..

[13] Nazir A., Arshad A.B., Lin S., Jeng J. (2022). Mechanical Performance of Lightweight- Designed Honeycomb Structures Fabricated using Multijet Fusion Additive Manufacturing Technology. 3D Print. Addit. Manuf..

[14] Kim H. (2002). New extruded multi-cell aluminum profile for maximum crash energy absorption and weight efficiency. Thin-Walled Struct..

[15] Dionisius F., Istiyanto J., Sumarsono D.A., Prayogo G., Baskoro A.S., Malawat M. (2022). Modeling of crashworthiness criteria based on variation of hole as crush initiator in thin-walled square. Int. J. Automot. Mech. Eng..

[16] Cheng Q., Altenhof W., Li L. (2006). Experimental investigations on the crush behaviour of AA6061-T6 aluminum square tubes with different types of through-hole discontinuities. Thin-Walled Struct..

[17] Bhutada S., Goel M.D. (2023). Progressive axial crushing behaviour of Al6061-T6 alloy tubes with geometrical modifications under impact loading. Thin-Walled Struct..

[18] Samer F., Tarlochan F., Samaka H., Khalid K., Abdulqadir S. (2013). Improvement of Energy Asorption of Thin Walled Hexagonal Tube Made of Magnesium Alloy by Using Trigger Mechansims. Int. J. Res. Eng. Technol..

[19] Hongyong J., Yiru R., Binhua G., Jinqu X., Fuh-Gwo Y. (2017). Design of Novel Plug-Type Triggers for Composite Square Tubes: Enhancement of Energy- Absorption Capacity and Inducing Failure Mechanisms. Int. J. Mech. Sci..

[20] Ren Y., Jiang H., Liu Z. (2019). Evaluation of Double- and Triple- Coupled Triggering Mechanisms to Improve Crashworthiness of Composite Tubes. Int. J. Mech. Sci..

[21] Lau S., Said M., Yaakob M. (2012). On the Effect of Geometrical Designs and Failure Modes in Composite Axial Crushing: A Literature Review. Compos. Struct..

[22] Altenolf B.A.W. (2010). Experimental observations on the crush characteristics of AA6061 T4 and T6 structural square tubes with and without circular discontinuities. Int. J. Crashworthiness.

[23] Reid S.R. (1985). Metal Tubes as Impact Energy Absorbers. Metal Forming and Impact Mechanics.

[24] Rezvani M., Jahan A. (2015). Effect of Initiator, Design, and Material on Crashworthiness Performance of Thin-Walled Cylindrical Tubes: A Primary Multi-Criteria Analysis in Lightweight Design. Thin-Walled Struct..

[25] Choi Y., Wereley N. Energy Absorption Efficiencies of Honeycomb Structures with Buckling Initiators. Proceedings of the AIAA SCITECH 2024 FORUM.

[26] Murray C., Wise S., Wereley N. (2024). Electroplating Additively Manufactured Honeycomb Structures to Increase Energy Absorption Under Quasi-Static Crush. SAMPE J..

[27] Murray C.M., Mao M., Park J., Howard J., Wereley N.M. (2023). Visco-Elastic Honeycomb Structures with Increased Energy Absorption and Shape Recovery Performance Using Buckling Initiators. Polymers.

[28] Timoshenko S., Gere J. (1961). Theory of Elasticity Stability.

[29] Roark R., Young J. (1975). Formulas Stress and Strain.

[30] Wierzbicki T. (1983). Crushing Analysis of Metal Honeycombs. Int. J. Impact Eng..

[31] Gibson L., Ashby M. (1997). Cellular Solids: Structures and Properties.

